# Collectanea Medica, Consisting of Anecdotes, Facts, Extracts, Illustrations, Queries, Suggestions, &c.

**Published:** 1819-03

**Authors:** 


### COLLECTANEA MEDICA,

CONSISTING OF

### ANECDOTES, FACTS, EXTRACTS, ILLUSTRATIONS, QUERIES, SUGGESTIONS, &c.

RELATING TO THE

*History or the Art of Medicine, and the Auxiliary Sciences.*

Quicquid agunt medici, nostri farrago libelli.

### *Medical Knowledge of the People of the Tonga Islands.*

*(Continued from page* 141.*)*

GITA (tetanus) is a disease very common among the Tonga people; but still more so among those of the Fiji islands, who, from their warlike habits, are more frequently in the way of it: they adopt, however, a remedy which the Tonga people have borrowed of them, and consists in the operation of *tocolósi*, or passing a reed first wetted with saliva into the urethra, so as to occasion a considerable irritation and discharge of blood; and, if the general spasm is very violent, they make a seton of this passage, by passing down a double thread, looped over the end of the reed; and, when it is felt in the perinæum, they cut down upon it, seize hold of the thread, and withdraw the reed, so that the two ends of the thread hang from the orifice of the urethra, and the doubled part from the artificial opening in the perinæum; the thread is occasionally drawn backwards and forwards, which excites very great pain and abundant discharge of blood. The latter operation Mr. Mariner has seen performed several times; but only twice for tetanus, arising in both instances from wounds in the foot: in these cases the spasms, but particularly the convulsive paroxysms, were exceedingly violent, extending to the whole body, neck, face, trunk, and extremities: but in neither case was the jaw permanently locked, though on every case it was violently closed for a few seconds. A native of the Fiji islands performed one operation, and Hali Ápi Ápi the other: they both happened at Vavaoo, at different times. In either case the disease came on suddenly, three or four days after the wound was received, which was from an arrow not barbed. The moment the symptoms became evident, *tocolósi* was performed. In the short space of two hours, one of them was greatly relieved, and the other in about six or eight hours. The following day, the one on whom Hala Ápi Ápi operated was quite well, and afterwards had no other attack; consequently the thread was withdrawn: but the other, on the second day, was not quite free from spasmodic symptoms, and a paroxysm coming on, the seton was moved frequently, which in two or three hours gave him great relief, and he afterwards had no other attack; it was thought prudent, however, to keep in the seton till the fourth or fifth day, when it was withdrawn. The effect of this operation was a considerable pain and tumefaction of the penis, but which gradually subsided in about five or six days. The artificial openings in both cases healed spontaneously, without any difficulty.

These are the only two cases of tetanus, in which this operation was performed, that Mr. Mariner can speak of with certainty, having been an eye-witness of them. He heard of several others at the Hapai islands, at the island of Tonga, &c. some of which were equally fortunate. From what he has heard and seen of the success of this operation at the Tonga islands, he is disposed to believe that about three or four in ten recover by the aid of it. The Fiji islanders, however, speak of the happy effects of this singular mode of cure with much more confidence than the natives of Tonga; but, as they claim the merit of the discovery, they are probably rather too profuse in praise of it.

Tetanus is not the only disease for the cure of which the operation of *tocolósi* is performed: it is adopted also in cases of wounds in the abdomen, upon the mistaken notion that any extravasated blood in the cavity of the abdomen is capable of passing off by the discharge from the urethra. Mr. Mariner saw the operation performed once in this case; and, as the man was considered in a very bad state, and notwithstanding got well, the cure was attributed to this remedy. It is also performed for relief in cases of general languor and inactivity of the system; but, in such instances, they only endeavour to produce irritation by passing the reed without any thread or artificial opening. The present king had it thus performed on him for this purpose, and two days afterwards he said he felt himself quite light and full of spirits.

The natives of these islands are very subject to enlarged testicles, and for this they sometimes perform the operation of *boca* (castration). Mr. Mariner's limited observation on this subject does not authorize him to speak with any degree of certainty in regard to the precise nature of these tumefactions. Their mode of performing this operation is summary enough. A bandage being tied with some degree of firmness round the upper part of the scrotum, so as to steady the diseased mass, at the same time that the scrotum is closely expanded over it, an incision is made with bamboo just large enough to allow the testicle to pass; which being separated from its cellular connexions, the cord is divided, and thus ends the operation. They neither tie the cord, nor take any pains to stop the bleeding; but, if the testicle be not very large, and the epidydimis not apparently diseased, they perform the operation by dissecting it from that body with the same instrument. The external wound is kept from closing by a pledget of the banana leaf, which is renewed every day till the discharge has ceased, and the scrotum is supported by a bandage. A profuse hæmorrhage is mostly the consequence of this operation. It was performed seven times within the sphere of Mr. Mariner's knowledge, during his stay, to three of which he was a witness: not one of the seven died. One of these cases was that of a man who performed the operation on himself: his left testicle was greatly enlarged, being about five or six inches in diameter, and gave him at times severe lancinating pains. Two or three times he was about to have the operation performed by a native of Fiji, but his courage failed him when he came to the trial. One day, when Mr. Mariner was with him, he suddenly determined to perform the operation on himself; and it was not much sooner said than done. He tied on the bandage, opened the scrotum with a very steady hand, in a fit of desperation divided the cord and cellular substance together, and fell senseless on the ground: the hæmorrhage was very profuse. Mr. Mariner called in some persons to his assistance, and he was carried into a house, but did not become sensible for nearly an hour, and was in a very weak state from loss of blood: this affair confined him to the house for two or three months. There was one rare instance of a man, both of whose testes were affected with some species of sarcoma, to a degree almost beyond credit. When he stood up, his feet were necessarily separated to the distance of three quarters of a yard, and the loaded scrotum, or rather the morbid mass, reached to within six inches of the ground. There was no appearance of a penis, the urine being discharged from a small orifice about the middle of the tumor,—that is to say, about a foot and a half below the os pubis. The man's general health was not bad; and he could even walk by the help of a stick, without having any sling or support for his burden: it was specifically lighter than fresh water, and considerably lighter than salt water, so as to produce much inconvenience to him when he bathed. He died at the island of Foa, about two or three months before Mr. Mariner left Vavaoo.

As to fractures, and dislocations of the extremities, it may be said that there is scarcely any native but what understands how to manage at least those that are most likely to happen; for they are very well acquainted with the general forms of the bones, and articulations of the extremities. They use splints made of a certain part of the cocoa-nut tree; for broken arms they use slings of gnatoo. In fractures of the cranium, they allow nature to take her course without interfering, and it is truly astonishing what injuries of this kind they will bear without fatal consequences. There was one man whose skull had been so beaten in, in two or three places, by the blows of a club, that his head had an odd mis-shapen appearance; and yet this man had very good health, except when he happened to take cava, which produced a temporary insanity. Fractures of the clavicle and ribs Mr. Mariner never saw there.

### *On Anthropophagism.* (From the *Dictionnaire des Sciences Medicales.)*

ANTHROPOPHAGISM (that horrible disposition of certain individuals, and even of whole nations, to employ the flesh of their own species as food,) has often excited the reflections of physicians and moralists, who have wished to discover the sources of so revolting an aberration from humanity.

Famine has been, without doubt, one of the most frequent, and most excusable, causes of anthropophagism. The Arabian ABDALLATIF has left us a frightful picture of the effects of a famine in Egypt: the putrefied remains of animals, . . . . . ., all the most disgusting of objects, were devoured with avidity. The want of food became so urgent, that flesh was torn from dead human bodies; children were strangled by their parents, for the purpose of feasting on their flesh; and bands of express anthropophagists traversed the whole country. It is only necessary to consult the histories of shipwrecks, in order to ascertain to what an extent excessive want may change the character and moral disposition of man; and, to remove all doubt of the existence of such horrors, civilized and, till then, sensible beings will be seen to have attended to nothing but the impetuous desire to calm the torments of famine, and have determined, by the indications of chance, which of their companions shall serve as food to the survivors.

A love of revenge has sometimes rendered men anthropophagists; and, although we may suppose that famine originally led hordes of cannibals to devour their prisoners, it cannot be denied that the fury of vengeance has, at least, perpetuated this crime amongst them.

I go to fight to revenge the loss of our brave fellows that were slain; I will be as merciless as the famished wolf; I will exterminate and devour our enemies; the tanned skins of their battered skulls shall hang in my dwelling; I will crush their wives and children, like the fearful storm of pouring hail; like the mighty thunder, will I consume their devoted villages:—such is an ancient war-song of the savages of Louisiana, and such is generally the spirit of those of all nations of cannibals: the battle-shout of the Iroquois is—"*We go to devour our enemies!*"

Religious opinions have also had some influence in producing the horrible custom of which we treat. Human sacrifices were in use among several ancient nations; and it was not strangers alone who were immolated, since parents delivered up the dearest objects of their affection, their own children, to the knife of the sacrificer. There is but one step from such an atrocious practice to that of anthropophagism; and the history of an almost-civilized people, the Mexicans, who, wanting none of the necessaries of life, regaled themselves with the flesh of the human victims that were offered to their idols, proves that in them, at least, it was not famine that gave rise to such odious banquets.

A fourth and last cause of anthropophagism especially demands the attention of the physician and the legislator: it consists in a depravation of the will, which has become subjugated to a depraved appetite. Although examples of such a deplorable state are very rare, yet modern times have furnished us with several such instances; amongst which, that of an anthropophagist of the environs of Wilna, the history of which was published by Professor Gruner, of Jena, is one of the most remarkable.*

John James Goldschmidt, a cow-herd, was married at the age of twenty-seven, and continued to follow the above-mentioned occupation for twenty-eight years, without any other moral vices being remarked in him than a certain rudeness and grossness of manners, and a great inclination to violent anger. He was fifty-five years of age in the year 1771, when great part of Germany was oppressed with famine: this circumstance, however, cannot be considered to have influenced the crime of Goldschmidt, which was committed in a fit of passion, since he had obtained provisions the day previously to it; he was not involved in debts, and his yard was stocked with poultry. This unfortunate man met with a young traveller at the entry of a forest, whom he reproached with having worried his cattle; the stranger asserted that he had not done this: a dispute and contest followed, and Goldschmidt killed the traveller by a blow with his stick. In order to conceal this circumstance from the public, he dragged the body of his victim into a thick part of the wood, cut it into small pieces, and carried one of them with him in his bag every time he returned to his dwelling. It was in one of those journeys that the desire to taste human flesh first developed itself in him: he boiled and roasted some part, and ate of it, with his wife, to whom he described it as mutton. A year afterwards, he enticed a child into his house, killed, and ate part of it. The crime was discovered, and it was from the confessions of the culprit that the details we have given were obtained, besides many others still more revolting, which we have thought proper to pass over in silence.

This fact led Hermann Gruner to form some very judicious reflections on this subject, with an abstract of which we shall terminate this article.

However criminal murder may be, particularly when it is followed by anthropophagism, the reality of a state cannot be contested where this has been induced by a depraved appetite, independent of the will. Nothing is more astonishing than the caprices of pregnant women, whom an insatiable inclination leads to enjoy with delight the most disgusting objects. Here, a fault of education, or the empire of a perverse habit, cannot be accused as the cause of such an aberration. The most serious remonstrances have no effect when advanced in opposition to an

* *De Anthropophago Bercano*, &c. in 4to.; *Diss. Inaug. Med. Resp.* F. G. A. Jacobi. Jenæ, 1781.

inexplicable desire, the satisfaction of which is demanded by nature in the most urgent and irresistable manner. May not this be equally applicable to the appetite of some individuals for human flesh? Whatever may have been the original cause of it, it depends principally on a disordered imagination: the appetite once indulged, it becomes increased, and at length habitual.

Many other criminals, besides Goldschmidt, have conceived this ardent inclination for feeding on human flesh only after having committed homicide. This disposition has also several times appeared in women in the state of pregnancy; an horrible proof of which is related by Abdallatif. At other times, anthropophagism has been observed as a family-evil: Hector Boëthius relates an instance of this in his History of Scotland. A Scotch free-booter, his wife, and children, were condemned to be burned for having drawn several persons to their dwelling, murdered, and devoured them. The youngest daughter was, however, exempted from this sentence, in consequence of her tender age; but she had hardly attained her twelfth year, when, from having perpetrated the crime of her parents, she was submitted to the same punishment.—"Why do you express disgust at my conduct?" said this young monster to the spectators, who were testifying their horror and detestation; "if you knew how delicious human flesh was, you would all eat your own children!"

Let us conclude with Dr. Gruner, that such desires, the idea alone of which should make the most insensible of men tremble with horror, ought really to be considered as a state of disease; and that physicians should thus interpret them to the expounders of criminal law.

---

*Relation of the Case of a Woman who voided the Bones of a Fœtus out of the Side of the Abdomen*; by Dr. Clarke.—MS. *Bibl. Sloan.* 698, Pl. xviii. F.; in the library of the *British Museum.*

In the year 165–, hearing by common report of a woman living in Denman's-alley in Drury-lane, that had carried a childe eighteen years in her belly, and now excluded the bones of it, after an extraordinary manner, by an abscess in the side of her belly, my curiosity, among others, carried me to see her. I found her a woman of seven or eight and forty years; tall, strongly made, and appearing to have been of a good habit of body; she had been now some years a widow, getting her own and her daughter's livelihood she had of about eleven years of age, by hard labour, going abroad to washing and scouring and such kind of work, until within some few weeks past; when she had lived by the charity of those whose curiosity brought them to see her.

I saw that she had two or three holes, from an abscess in the left side of the abdomen, near the place where the incision for a ——— is usually made, out of which came matter and small bones, which had been the vertebres of a childe. I saw divers others she then had in a box, being several of the bones of a scull, the shoulder-blades, ribs, vertebres, bones of the limbs, which had all been excluded from those holes in the abscess, and were the bones of a perfectly-formed childe; of which she every day excluded yet more. I had from herself the following story:—

That, about eighteen years before, having after, as she thought, gone her full time with childe, she endured two or three days of strong pain, without any hopes of being delivered; when one of the women, to give her ease, with a large towel compassing the bottom of her belly, lifting it up, standing behind her, upon which she felt some part, as she thought, break within her, and something removed from the bottom of her belly into her side. From this time she felt no more pain, and by degrees, as she thought, grew well: the swelling of her belly something diminishing, though continuing always considerably swelled in the left side.

After two years, or thereabout, she conceived, and in due time brought forth a female childe, which lived about two or three years: but after four years more, or thereabout, she brought forth another female childe, which was the daughter I then saw with her. She enjoyed her health well, until some few months before I saw her; when, having a great pain on that part of her belly, which continued swelled, she received from some one an oyntment, upon use of which, now being all inflamed, she often used poultices to the swelled part, which breaking and a great deal of matter running out. After some weeks, she found bones offer themselves to come out at the surface; and from that time, every day for the most part, took out some, until I think she had all the bones of a perfectly-formed childe.

---

"Æmilius" would be obliged to any of our readers who would inform him if experiments have been made to ascertain the cause why blood drawn from a vein of a person who has been in a warm bath for ten minutes or longer, is of a florid red colour,—i. e. similar in appearance to arterial blood? and, also, why the blood which flows during the latter stage of the ordinary operation of blood-letting from the arm, has likewise a florid appearance? He, himself, imagines this to arise from suspension of the functions of the secretory capillary vessels; but would feel grateful for a reply to the above queries, or for any theoretical opinions respecting the cause of these phenomena; and also whether the notion he has advanced respecting it will not explain some of the effects witnessed from the use of the warm bath.

---

In the "*Philosophical Transactions*" for the year 1709, there is a relation of the case of a girl, sixteen years of age, who, about the end of April (1709), had some red pimples appear on her face, which subsided after bleeding and some purgative medicines. She continued very well until about a month afterwards, when her face, "so far as is usually covered with a vizard-mask, suddenly turned black like that of a *negro*." Mr. J. Yonge, who relates the case, directed a lotion for her face, which removed the discoloration; "yet it returned frequently, but with no regularity, sometimes twice or thrice in twenty-four hours, sometimes five or six times. It appears insensibly, without pain, sickness, or any symptoms of its approach, except a little warm flushing just before it appears. It easily comes away, and leaves the skin clear and white, but smuts the cloth that wipes it from the face; it feels unctuous, and seems like grease and soot, or blacking, mixed; it has no taste.—She never had the *menses;* is thin, but healthful. The blackness appears no-where but in the prominent part of her face. It is now (Nov. 1) divided into a few dark cloudy specks, which appear but seldom, and not so livid as formerly."

### *Cassia Marilandica* (American Senna). (From Dr. Barton's *American Medical Botany.)*

CASSIA. *Cal.* 5, phyllus; *petala,* 5; *antheræ* supremæ, 3, steriles; infinæ, 3, rostratæ. *Lomentum,* (Willdenow).

Nat. Syst. Jussieu, *Leguminosæ*; classis xiv. ordo xi.

Nat. Ord. Lin. *Lomentaceæ*; classis *Decandria,* ordo *Monogynia.*

——— Marilandica. C. foliis octojugis ovato-oblongis æqualibus, glandula baseos petiolorum. Willd.

*Obs.* Foliola mucronata. Legumen angusto-lineare, arcuatum, glabrum. Mich. Fl. Bor.

The generic name of this plant is of Asiatic origin, and was brought into Greece along with the commercial article which it denoted, by the Phœnician merchants.* The specific appellation was given by Linnæus, in conformity with a common custom, (of which later discoveries have shown the impropriety,)—that of naming a new species of any genus from the particular place whence it was sent to him. Though the first specimens of Cassia Marilandica were transmitted to Linnæus from the state of Maryland, the plant is now known to be extremely common in almost every state of the Union south and westward of New-York. Inappropriate as the specific name is, however, it still does, and always should stand, unchanged.

The wild, or American, senna is a beautiful plant. It is about three or four feet high, with stems rising erectly from the root. The root is perennial, mostly horizontal, but sometimes perpendicular; contorted, irregularly shaped, woody, black, and covered with a multitude of fibres, also of a black colour externally, and yellow within. The stems many, often herbaceous, cylindrical, either entirely smooth or furnished with a few hairs. The leaves are alternate, rather long, green above, and pale underneath. Leaflets in eight pairs, ovate oblong, equal, and yellow on the margin; a gland at the base of the petioles. Flowers bright

* It is the קציעה of the Hebrews and other Orientals.

orange-yellow, in short axillary racemes, on the upper part of the stem. Legumes three or four inches long, a little curved, mucronate, bordered with conspicuous joints, and a few scattered reddish hairs.

This plant is pretty common, from New-York to Carolina, and, where met with, is generally abundant. Though it sometimes is found remote from water, it will always, I think, appear, on examination, that such situations are exsiccated swamps or meadows. It delights in a low, moist, gravelly or sandy soil, preferring the borders of rivers, creeks, and such watery places, to any other situations; and flowers from the last of June to the last of August.

*Medical Properties.*—Wild senna is now well known to be a valuable cathartic of the milder class. It is little, if at all, inferior to the senna of the shops.* Professor Hewson, of Philadelphia, informed me that he had used it occasionally, and with the same good effect as common senna; and I have had some experience with it in my own practice. At the Marine Hospital of the navy-yard, I have for some months past substituted it for Alexandrian senna, and have had reason to confirm the high character of the plant, which it has long maintained. The leaves alone have commonly been used; but I have employed the dried leaves and follicles, carefully rejecting the leaf-stalks, and recommend this manner of using the plant for medicinal purposes. I believe the best time for collecting it would be when the pods are ripe, which is about the latter end of August.

Since it appears that we do not obtain pure senna from Egypt, and that the adulterating plant, or *cassia senna*, is much inferior to our native species, it cannot be doubted that an admixture of the cassia lanceolata and cynanchum oleæfolium (which are the plants mixed with the true senna in Egypt, according to M. Nectoux,) with the cassia Marilandica, would afford a much better article than that now used, and at one-fourth of the price.†

* It appears by the researches of M. H. Nectoux, that botanists and writers on materia medica have hitherto erred in supposing the true senna of the shops to be the leaves and follicles of the cassia senna of Linnæus. This intelligent and industrious enquirer instituted in Egypt a series of investigations respecting the senna, which resulted in the ascertainment of the fact that cassia senna, L. is in reality a weed, with which the real senna is adulterated in Egypt.

† The cassia Marilandica was introduced into England in 1723, by Peter Collinson, esq. It flowers here from August to October. Specimens of it are preserved in Kew-garden.

